# Osteoregenerative Potential of 3D-Printed Poly *ε*-Caprolactone Tissue Scaffolds In Vitro Using Minimally Manipulative Expansion of Primary Human Bone Marrow Stem Cells

**DOI:** 10.3390/ijms24054940

**Published:** 2023-03-03

**Authors:** Logan M. Lawrence, Roozbeh (Ross) Salary, Virginia Miller, Anisha Valluri, Krista L. Denning, Shannon Case-Perry, Karim Abdelgaber, Shannon Smith, Pier Paolo Claudio, James B. Day

**Affiliations:** 1 Department of Pathology, Joan C. Edwards School of Medicine, Cabell Huntington Hospital Laboratory, Marshall University, Huntington, WV 25701, USA; 2 Department of Mechanical Engineering, Marshall University, Huntington, WV 25703, USA; 3 Department of Biomedical Engineering, Marshall University, Huntington, WV 25755, USA; 4 Joan C. Edwards School of Medicine, Marshall University, Huntington, WV 25701, USA; 5 Cabell Huntington Hospital Laboratory, Department of Histology, Mountain Health Network, Huntington, WV 25701, USA; 6 Department of Pharmacology and Toxicology, University of Mississippi Medical Center, Jackson, MS 39216, USA; 7 Department of Maxillo-Facial Surgery, University of Mississippi Medical Center, Jackson, MS 39216, USA; 8 Department of Orthopaedic Surgery, Joan C. Edwards School of Medicine, Marshall University, Huntington, WV 25701, USA

**Keywords:** 3D microfabrication, autograft, allograft, bone graft, bone marrow, bone regeneration, tissue scaffold, stem cells

## Abstract

The repair of orthopedic and maxillofacial defects in modern medicine currently relies heavily on the use of autograft, allograft, void fillers, or other structural material composites. This study examines the in vitro osteo regenerative potential of polycaprolactone (PCL) tissue scaffolding, fabricated via a three-dimensional (3D) additive manufacturing technology, i.e., a pneumatic micro extrusion (PME) process. The objectives of this study were: (i) To examine the innate osteoinductive and osteoconductive potential of 3D-printed PCL tissue scaffolding and (ii) To perform a direct in vitro comparison of 3D-printed PCL scaffolding with allograft Allowash^®^ cancellous bone cubes with regards to cell-scaffold interactions and biocompatibility with three primary human bone marrow (hBM) stem cell lines. This study specifically examined cell survival, cell integration, intra-scaffold cell proliferation, and differentiation of progenitor cells to investigate the potential of 3D-printed PCL scaffolds as an alternative to allograft bone material for the repair of orthopedic injuries. We found that mechanically robust PCL bone scaffolds can be fabricated via the PME process and the resulting material did not elicit detectable cytotoxicity. When the widely used osteogenic model SAOS-2 was cultured in PCL extract medium, no detectable effect was observed on cell viability or proliferation with multiple test groups showing viability ranges of 92.2% to 100% relative to a control group with a standard deviation of ±10%. In addition, we found that the honeycomb infill pattern of the 3D-printed PCL scaffold allowed for superior mesenchymal stem-cell integration, proliferation, and biomass increase. When healthy and active primary hBM cell lines, having documented in vitro growth rates with doubling times of 23.9, 24.67, and 30.94 h, were cultured directly into 3D-printed PCL scaffolds, impressive biomass increase values were observed. It was found that the PCL scaffolding material allowed for biomass increase values of 17.17%, 17.14%, and 18.18%, compared to values of 4.29% for allograph material cultured under identical parameters. It was also found that the honeycomb scaffold infill pattern was superior to the cubic and rectangular matrix structures, and provided a superior microenvironment for osteogenic and hematopoietic progenitor cell activity and auto-differentiation of primary hBM stem cells. Histological and immunohistochemical studies performed in this work confirmed the regenerative potential of PCL matrices in the orthopedic setting by displaying the integration, self-organization, and auto-differentiation of hBM progenitor cells within the matrix. Differentiation products including mineralization, self-organizing “proto-osteon” structures, and in vitro erythropoiesis were observed in conjunction with the documented expression of expected bone marrow differentiative markers including CD-99 (>70%), CD-71 (>60%), and CD-61 (>5%). All of the studies were conducted without the addition of any exogenous chemical or hormonal stimulation and exclusively utilized the abiotic and inert material polycaprolactone; setting this work apart from the vast majority of contemporary investigations into synthetic bone scaffold fabrication In summary, this study demonstrates the unique clinical potential of 3D-printed PCL scaffolds for stem cell expansion and incorporation into advanced microstructures created via PME manufacturing to generate a physiologically inert temporary bony defect graft with significant autograft features for enhanced end-stage healing.

## 1. Introduction

### 1.1. Review of Literature

#### 1.1.1. Human Bone Regeneration

In the United States, bone grafts are the most common tissue transplants and are a key tool used in reconstructive maxillofacial and orthopedic surgery [1]. The current mainstream treatment in reconstructive orthopedic surgery remains autograft tissue, which is harvested, for example, as either cortical (strut grafts), cancellous (iliac crest), or bone marrow material taken from the patient, and re-implanted at the site of injury. Iliac crest bone graft (ICBG) is generally accepted as the gold standard for autograft [2,3,4,5,6,7,8]. However, these treatment options can be limited due to the general lack of viable material for sizable defects and donor-site morbidity [5,6]. In such cases, “bone void fillers” (BVF) are often employed. A prime example of a BVF is allograft decellularized bone material (DBM), which are often used as a viable option where autograft tissue is insufficient or is otherwise not available. The advancement in *allogenic* bone grafting over the past decades has progressed away from the necessity of autologous transplantation for the repair of a variety of traumatic reconstructive procedures. The use of allograft material, however, is not without its own set of drawbacks. These include availability, sterilization, cost, immune reaction, delayed graft incorporation, disease transmission, and long-term graft stability and remodeling [2,3,4,5,6,7,8]. Consequently, artificial bone substitutes have been in development, but the inorganic nature of these materials raises concerns about durability. Thus, materials of this sort will have to address their permanency and end-state resemblance to the bone in terms of remodeling potential and natural durability [9].

The mechanical and biological functions of human bones are reliant on the specific architecture, location and function, and as such healthy bone serves as a living and constantly remodeling somatic support framework for the body [7]. The mechanical properties of the bones, which allow for efficient movement and attachment of muscles and connective tissue, are designed to perform specific functions throughout the body and are generally defined by their tensile, compressive, and bending strengths, as well as fracture toughness. In the long bones of the human body, the porosity of the bony macrostructure and microstructure (collagen and hydroxycarbonate apatite) in the outer cortical bone ranges from 10–30%. The porosity increases in the inside of the bone (cancellous bone) to an average of 30–90% depending on the specific function and location of the bone [10]. Some bones, such as the ribs, serve functions reliant on a degree of elasticity and tensile strength, while other bones, such as the femur and tibia, are reliant on compressive strength and load bearing.

In general, structure and porosity play a significant role in the functional performance and integrity of fabricated bone scaffolds. In addition, a wide range of porous micro-structures with various internal morphologies (such as honeycomb, rectilinear, and concentric [11], as well as triply periodic minimal surfaces [12,13,14,15], which have the potential to significantly enhance the mechanical performance of bone scaffolds) can be additively fabricated for bone tissue engineering. Too small pores not only prevent cell migration but also limit the diffusion of nutrients and removal of waste products. Additionally, closed pores hinder transport of materials, proliferation of cells, as well as osteoconduction within a fabricated scaffold. Pores that are too large result in a decline in the surface area required for cell adhesion; they also lead to a decrease in the mechanical properties [16], but improve cellular infiltration. Therefore, fabrication of scaffolds with optimal porosity is an unavoidable medical need while working towards durability [17]. Thus, healthy bone is generally divided into heterogeneous macrostructures of cancellous and cortical bone, and the microstructures such as individual trabeculae and osteons, cellular structures, and mineral and nutrient components (minerals and collagen). In summary, skeletal bone has a complex and highly organized structure and multiple functions within the body [5,7,9,10]. 

In terms of fracture healing, bone can undergo two generally broad categories of normal repair—healing (primary) and/or enchondromal healing (secondary). These healing pathways form a unique characteristic of bone repair in that when complete, it is nearly indistinguishable from the embryogenic original bone, and not substitutive or “scar” tissue [10,18]. In fracture healing, the body will repair the injured bone through one of these mechanisms. Primary healing is typical for simpler fractures without defects, while secondary healing is more common and typical in segmental defect and surgical stabilization repair. Of particular interest here is secondary healing in the setting of large defects. Secondary bone healing is largely a four-staged process—first with inflammatory response and hematoma formation, followed by clotting of blood and fibrin at the fracture site to serve as a foundation for the formation of new bone. The clot is slowly replaced by collagen and fibrotic connective tissue to form a soft disorganized “callus”. The callus, which is composed of connective tissue and cartilage with embedded mesenchymal stem cells (MSCs), will slowly harden as the cells differentiate into mature, osteoblasts, and eventually osteocytes as this matrix is calcified [18,19]. Thus, hBM stem cells normally undergo differentiation in healthy bone [7,19]. Primary healing is not typically feasible in bony defects without some form of grafting (i.e., an intercalary or strut graft), and as such generally requires a prolonged period of stabilization for the graft to undergo successful incorporation and healing [18,19]. Since secondary healing occurs through the staged process described above, and typically requires less rigid fixation the bone heals through a process where more of the aforementioned grafting techniques are appropriate. Furthermore, micromotion in secondary healing is considered critical for the healing of bone to the normal end-state—too much motion leads to the “hypertrophic non-union” while too little leads to the so-called “atrophic” non-union. Considerable fabrication studies have been performed aiming to make “durable” artificial scaffolds, which have studied the optimal structure for mechanical strength, but empiric optimization based on microstructure geometry for cellular incorporation appears to be limited, particularly using PCL alone without additives. It is worth mentioning that all bone healing should be considered to heal through some combination of these two processes [20]. In any case, successful healing requires that a suitable fabricated graft should have the characteristics of being able to undergo extensive absorption and deposition of the scaffold to return to normal bone [19,21]. Hence, timing is critical for entry into the final stage of healing. As a result, in the case of missing bones or a fracture that has failed to completely elicit the healing response (atrophic non-unions), the missing portion of bone must be replaced with some form of functional equivalent, which ultimately leads to normal bone. Currently, such grafting takes the form of one of several broad groups: autograft (taken from the patient directly); allograft (bone grafting from donors other than the patient); xenograft (derived from other species); and, lastly, artificial or fabricated bone graft substitutes [2,3,6]. Furthermore, grafting material falls into several broad biologically active classifications: (i) osteogenic, (ii) osteoconductive, and (iii) osteoinductive activity. An osteogenic material is defined as a material that either contains a bony matrix or transforms into native bone while containing the prerequisite cellular material for bone formation. An example of this is an autogenous iliac crest bone graft, which is the current gold standard for bone grafting. The osteoconductive material promotes bone incorporation from the adjacent native bone through the recruitment of neighboring bone-forming cells and eventually transforming the allograft into host bone. An example of an osteoconductive bone graft would be allographic cancellous chips. An osteoinductive material stimulates local or transplanted cells to enter a pathway of native bone healing through cellular stimulation. A demineralized bone matrix would be an example [3,9].

Unfortunately, in addition to being limited, surgical techniques requiring large defect reconstruction often present additional operative co-morbidities and frankly can be quite painful (ICBG) [4]. Since these procedures are effective for repairing bony defects, it is no surprise that large defects or voids often pose more of a challenge for these surgical repairs [4,22]. The use of allograft decellularized donor bone tissue has emerged as a commonly employed BVF for larger voids in injured bone and is used frequently as a supplement to autograft to expand the volume. It is even commonly used as a stand-alone graft. However, as previously noted, allograft has drawbacks. These include, but are not limited to its availability, sterilization, cost, immune reaction, delayed graft incorporation, disease transmission, and challenges surrounding long-term graft stability and incomplete remodeling [4,22], and they provide little if any structural supplementation. In recent years, the processes of acquiring and sterilizing donor bone tissue for transplant have improved substantially, the risks and challenges however to its clinical utilization still remain [22]. Furthermore, the costs of such materials have risen with the increased sophistication of the sterilization and processing techniques now used in its production [23]. Other limiting factors of the use of allograft bone material are the storage requirements and relatively short expiration dates. This, coupled with the general lack of customizability on a patient-by-patient basis, makes the argument for a customizable BVD that can provide for rapid remodeling and a degree of structural support [4,22,23]. In the last twenty years, a significant advancement in segmental bone healing occurred with the so-called Mesquelet or “membrane” healing of bony defects [24,25,26]. This technique allows for secondary healing by stimulating increased angiogenesis before grafting. The Mesquelet technique, unfortunately, is a staged process itself, and the grafting step can be viewed as an *early* entry into the four staged process of secondary healing—thus essentially requiring the full time to heal and consequently the risk of the process arresting altogether [26]. To avoid the risks and limitations associated with tissue donated bone, there are many ongoing efforts to develop synthetic bone scaffolds which can be manufactured on a large scale resulting in cheaper and more customizable options to take the place of allograft, and even, to a degree, autogenous materials. When attempting to design an effective tissue substitute to replace bone, several factors must be considered: (i) overall biocompatibility and cell attachment; (ii) biodegradability and lack of toxicity; (iii) immunologically inertness and compatibility; (iv) mechanical properties and stability; (v) specific features of porosity to allow for nutrient exchange and angiogenesis; (vi) osteoinductive and potentially osteoconductive properties; (vii) and ease of sterilization and long term shelf-life [27,28]; (vii) be structurally similar to the gross architecture of the missing bone. The focus of our study has been to develop a low-cost, relatively mechanically stable, and biologically compatible manufactured bone graft substitute with micro and macrostructure more closely resembling native bone in the *late stages* of callus remodeling of large skeletal defect. This would represent a significant advance in both accelerated and endpoint healing of boney segmental defects.

#### 1.1.2. 3D-Microfabrication

The ongoing revolution in 3D printing technology in the manufacturing sector has allowed for a variety of software-based platforms to fabricate polymeric objects of infinite shapes and dimensions at a rapid pace [29]. Various additive manufacturing techniques have been successfully utilized for tissue engineering applications, such as fused deposition modeling (FDM), pneumatic micro-extrusion (PME), stereolithography (SLA), direct ink writing (DIW), laser-guided direct writing (LGDW), and inkjet bioprinting [30]. Additive manufacturing techniques allow for the creation of custom, complex, and porous microstructures with interconnected lattices [31] with great accuracy [32], as well as offering various biomedical advantages, such as the co-culture of multiple cells as well as the incorporation of growth factors [33].

**Figure 1:**
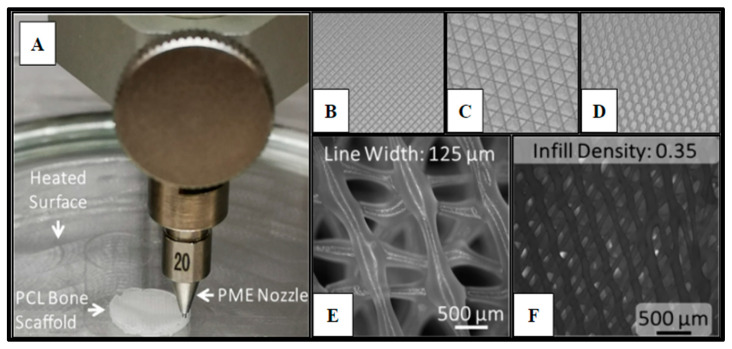
Polycaprolactone (PCL) 3D-printed scaffold fabrication. Panel (**A**) demonstrates the PME process for scaffold microfabrication. Panels (**B**–**D**) display the infill patterns for the tested scaffold designs: (**B**) Rectangular; (**C**) Cubic; (**D**) Honeycomb. Panel (**E**) presents the honeycomb line width parameter captured on a fluorescence microscope with a 2X magnification (B2X, Keyence, Itasca, IL). Panel (**F**) displays infill density.

Pneumatic micro-extrusion has emerged as a high-resolution method for the fabrication of a plethora of tissues and organs for clinical applications. PME has a layer resolution of 1 μm and has paved the way for the deposition of a wide range of functional bio-inks for regenerative engineering applications. Highly complex and nonlinear in material deposition, the PME process is governed by multi-physics phenomena, such as phase change. There is a wide range of PME process parameters, including but not limited to solidification rate, viscosity, flow temperature, as well as flow pressure. In the PME process, a high-pressure flow of air (usually supplied by an oilless compressor) is the medium of material transport and subsequently material deposition. The deposition head’s cartridge is loaded with a polymer material (such as PCL). The loaded polymer material is heated to a temperature above the polymer’s melting temperature. Finally, the molten polymer flow is deposited on a heated or cooled free surface with the aid of the high-pressure air flow using a micro-capillary nozzle (as exemplified in Figure 1A). Yu et al. [11,34,35] observed (on the basis of a computational fluid dynamics (CFD) model) that molten PCL flow through a nozzle—with a nozzle diameter and flow pressure of 200 µm and 550 kPa, respectively—was a viscous flow. The rate of material solidification (and subsequently layer adhesion) in the PME process can be, to a great extent, controlled using a filtered airflow, supplied at a constant rate to the chamber. There is a broad spectrum of natural-origin materials (such as chitosan, collagen, silk fibroin, starch in addition to calcium phosphate and silicate ceramics) [33], as well as synthetic polymers (such as polyesters of α-hydroxy acids and lactones, polyorthoesters, polycarbonates, and polyurethanes), available for regenerative medicine [30,31,32,36]. To enhance the printability of ceramics, they can be combined with polymers [33]. Poly(ε-caprolactone) (PCL), utilized in this work, is a semicrystalline and biocompatible polylactone. PCL has a relatively low degradation rate and high mechanical strength, which make PCL potentially suitable for not only cell-based therapies, but also fabrication of bone substitutes for rapid incorporation and eventually treatment of large defects (as end stage healing). Rodriguez et al. synthesized highly permeable poly(L-lactide-co-ε-caprolactone) for the regeneration of large gaps [37].

In addition to PCL, poly (lactic acid) (PLA), polyether ether ketone (PEEK), and poly vinyl alcohol (PVA) have emerged as potential biocompatible materials for regenerative engineering applications, which allow for FDM-based fabrication of biological tissues and constructs. Materials that are compatible with SLA process include but not limited to poly(D,L-lactide) (PDLLA), poly (propylene fumarate) (PPF), and poly(ethylene glycol) diacrylate (PEGDA), and gelatin-methacryloyl (GelMA) inks. Additionally, GelMA and hydrogel inks are suitable for DIW and LGDW additive manufacturing processes [30]. In addition to polymeric scaffolds, novel metal-based scaffolds (such as magnesium [38], iron [39], titanium alloy [40], and zirconia [41] scaffolds) have been fabricated for tissue engineering applications.

In a research work by Wang et al., cryogenic 3D printing was used for the fabrication of hierarchically porous bone tissue scaffolds, being both osteoconductive and osteoinductive, as required for bone regeneration [42]. Using angiogenic peptide and collagen type-I hydrogel, the fabricated scaffolds were given osteogenic and angiogenic capabilities. In addition, it was observed that the scaffolds were biocompatible (characterized based on rat bone marrow mesenchymal stem cells and rat endothelial cells). Chen et al. [43] successfully demonstrated additive fabrication of complex nano-fibrous scaffolds using a combined method including reverse solid freeform fabrication along with thermal phase separation with the aim to precisely control the external morphology and the internal microstructure of the fabricated scaffolds. Inzana et al. [44] explored low-temperature additive fabrication of calcium phosphate- and collagen-based bone scaffolds utilizing inkjet printing. The incorporation of collagen significantly improved the flexural strength in addition to the cell viability of the scaffolds and led to osteoconductivity. In a research work by Zhang et al. [45], hollow silicate bioceramic-based scaffolds (with high degree of vascularization) were fabricated based on 3D coaxial printing. Capable of both osteogenesis and angiogenesis, the fabricated scaffolds had a compressive strength of 26 MPa. Furthermore, the scaffolds were capable of rapid infiltration of host blood vessels in addition to delivery of not only growth factors but also stem cells. Biomimetic bone scaffolds (consisting of biomineralized hydroxyapatite nanocomposite, deposited on silk fibroin) were 3D fabricated. It was observed that the fabricated scaffolds had a compressive strength of approximately 6 MPa and a porosity of about 70% with enhanced cell proliferation and attachment. This study examines the potential for the production of PCL bone scaffolding fabricated via a novel pneumatic micro extrusion (PME) 3D printing technique, coupled with our previously published minimally manipulative human stem cell mesenchymal expansion technique [46]. Software technologies now exist to pair medical imaging data to 3D printers to enable the accurate recreation of patient-specific anatomical structures [29,47]. This merging of novel manufacturing techniques and clinical patient data may allow for the rapid fabrication of patient-specific 3D-printed tissue scaffolds for use in regenerative medicine and orthopedic and maxillofacial reconstructive surgery, in particular. Thus, if these structures could be optimally fabricated to enhance the incorporation of the MSCs and thus ultimately osteoblasts and osteoclasts, it would represent a significant advancement. Furthermore, a simple method of mesenchymal procurement can be utilized and the cells incorporated into the fabricated scaffolds a priori, such a material could be viewed as a fabricated structural autograft with features of all three biological classifications of bone graft and function at a late stage of bone healing. This study investigated the biocompatibility, osteoinductive, osteoconductive, and osteogenic potential of 3D printed PCL tissue scaffolds, with various microstructures, and without the use of additional bioactive additives in vitro as compared to allograft bone void filler.

## 2. Results

### 2.1. Polycaprolactone (PCL) 3D-Printed Scaffold Fabrication

Cell scaffolds of Polycaprolactone (Cellink, Boston, MA, USA) were fabricated (Methods 4.8) using a specialized pneumatic micro-extrusion (PME) process (Figure 1A).

The fabrication parameters and optimization, which have been detailed in previous studies [11,13,48,49], resulted in PCL scaffolds with varying infill designs, such as Honeycomb (HC), Rectilinear (RL), and Cubic (Cub) (Figure 1B–D), printed at infill densities of 0.35 and line width of 125 µm (Figure 1D–E). Each scaffold design utilized in this study met the consistent parameters of 10 mm in diameter and 3 mm in height. The ability to adequately control for and eliminate structural variability in the fabrication of the scaffolding materials utilized in this study is crucial to the potential of advancing this technology into the clinical setting and allows for scalability of the manufacturing process. Yu et al. observed that the Honeycomb pattern relatively had the highest amount of stiffness, followed by Rectilinear, Concentric, Cubic, and Gyrid [11].

### 2.2. Human Bone Marrow (hBM) Sample Collection

**Figure 2:**
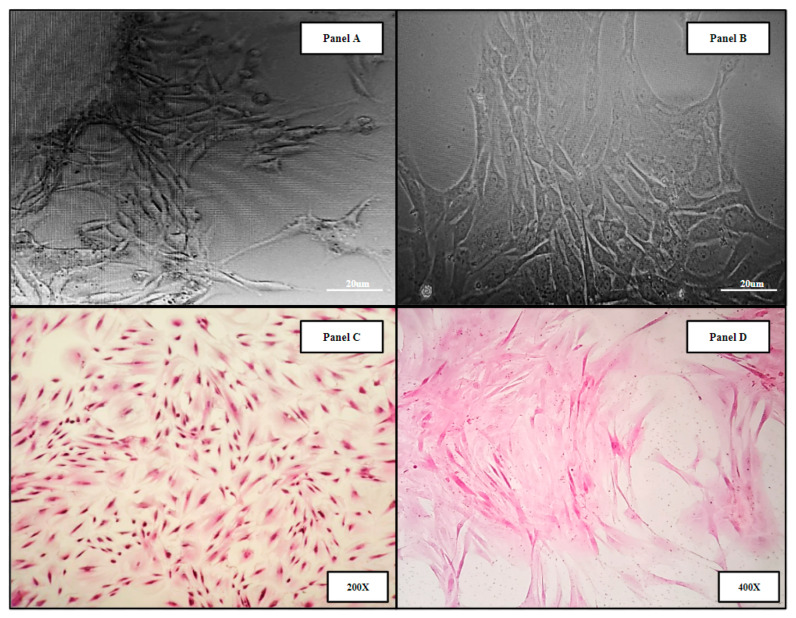
Representative images of primary hBM cell populations isolated from whole bone marrow aspirate and material from long bone fracture reaming. The derived cell populations are composed of native bone marrow progenitor cell subtypes including mesenchymal stromal cells and hematopoietic stem cells. Panels (**A**,**B**) display primary hBM cells in culture taken at days 5 and 15, respectively, as the populations were expanded. Panels (**C**,**D**) display monolayer H&E-stained slides of the hBM cell population.

Human bone marrow samples were obtained from four patients undergoing intramedullary nail fixation for closed femoral fractures (*Methods 4.1*). The resulting primary human bone marrow cell lines (hBM) BM006, BM008, and BM009 were used in the in vitro analysis of the 3D-printed and allograft scaffolds. Micrograph images of the derived primary cell lines can be seen in (Figure 2). Panels A,B show primary hBM cells in culture taken at days 5 and 15, respectively, as the populations were expanded. Panels C,D display monolayer H&E-stained slides of the hBM cell population. The cellular phenotypic characteristics are predominantly of the spindle cell variety consistent with mesenchymal stem cells. The cell populations isolated were completely cleared of non-adherent cells and debris, i.e., erythrocytes and residual boney material during initial processing. The complete clearing of differentiation products and native boney matrix components in the established primary (hBM) cell lines prior to scaffold seeding were crucial to this study, ensuring that any markers of differentiation detected in the final analysis were de novo and not the result of primary sample contamination.

We found that the reamer irrigator aspirator (RIA—see Section 4.1) method of reaming is less optimal for the collection of viable stem cells. Better proliferative characteristics were observed from the “non-washed” standard reaming collection even though the amount of material collected by this method is often limited. Of the four primary bone marrow samples collected for this study, three samples yielded sufficient viable mesenchymal stem cell colonies, which were expanded in vitro for further study (Methods, Section 4.5). One of the four samples, BM007, did not yield a large enough cell population and was excluded from the study. The effect of collection method on cell viability of the reaming material is of note due to the future implications of an intraoperative scaffold seeding methodology for orthopedic wound healing. Further donor site cell viability studies will be needed to investigate the potential of this approach in the clinical setting.

### 2.3. Primary Whole Bone Marrow Progenitor Cell Line Isolation and In Vitro Expansion

**Figure 3:**
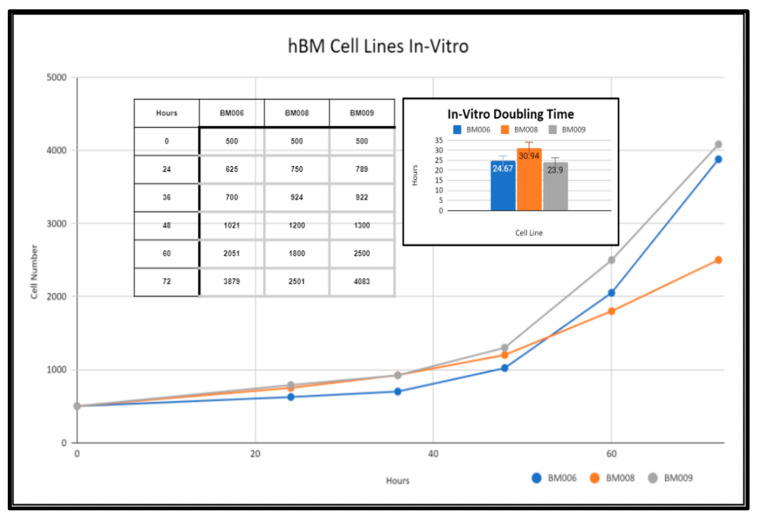
In vitro growth rates of primary hBM adherent cell cultures.

Primary human whole blood marrow adherent stem cell progenitor populations were isolated from whole marrow reaming samples via monolayer tissue culture techniques as detailed in the methods section (Methods, Section 4.5). The derived adherent cell cultures were not selected for specific subpopulations of progenitor cells and were composed of both mesenchymal and hematopoietic cells present within the native somatic niche within the femoral bone marrow. These primary cell cultures were expanded in vitro and displayed varying growth rates as reflected in Figure 3. Adherent primary stromal cell cultures were identified in culture 48 h after collection in samples BM006, BM008, and BM009. The adherent cell populations were cleared of any non-adherent cells and bone marrow material including red blood cells (RBCs), platelets, lipid aggregates, and bone fragments from the collections before investigations were performed in the study. After clearing the primary cultures of non-adherent material, in vitro growth rates of BM006, BM008, and BM009 were measured over a period of 72 h and displayed doubling times of 24.67, 30.94, and 23.9 h, respectively (Figure 3). These cultures served as in vitro models of primary human bone marrow (hBM) cell populations for in vitro osteoregenerative studies. The viability and overall health of the primary cell populations used in this study had a direct impact on the observed differentiation and integration potential of the primary stem-cells into the tested scaffolding materials. Prior to scaffold seeding, the primary stem-cell populations were mitotically active and did not exhibit signs of population quiescence or senescence.

### 2.4. Screening of Biocompatibility of Fabricated Polycaprolactone (PCL) Scaffolds via Cytotoxic and Cytostatic Analysis of PCL Extract Exposure and Histological Analysis of Established Osteosarcoma Cell Line SAOS-2

**Figure 4:**
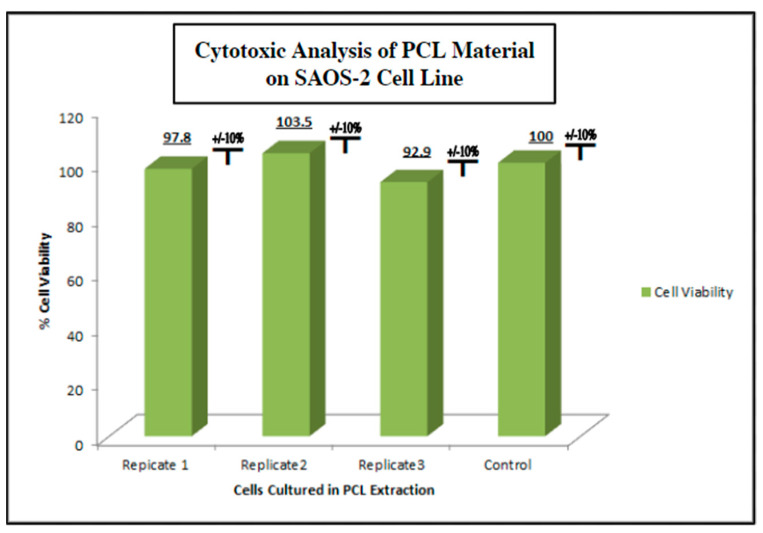
Cytotoxic/cytostatic screening of PCL material extract with SAOS-2 representative osteogenic cell line. SAOS-2 cells were cultured in PCL media extract and assessed for viability via MTT assay *n* = 8 (Methods, Section 4.4). No cytotoxic or cytostatic activity was noted when compared to the non-extract control group.

To measure the potential inhibition of cell growth in cells exposed to PCL extract on human cells under physiologic conditions, as in vitro model, we used the human osteosarcoma cell line SAOS-2 (ATCC) (Methods, Section 4.4). SAOS-2 cells were cultured in the PCL media extract for 48 h and evaluated for viability using an MTT assay (Figure 4). We observed no cytotoxic or cytostatic activity of the PCL media extract as compared to a control group cultured in a standard culture medium. The viability assay was run in triplicate with each test group consisting of 8 individual replicates. The viability average of each PCL extract group ranged from 92.9% to 100.0% viability compared to the control group with a standard deviation of ±10%. The lack of significant bioactivity or toxicity to possible PCL degradation products in the in vitro setting used for this study ensured that the inert nature of polycaprolactone reported in prior studies was confirmed in this setting.

**Figure 5:**
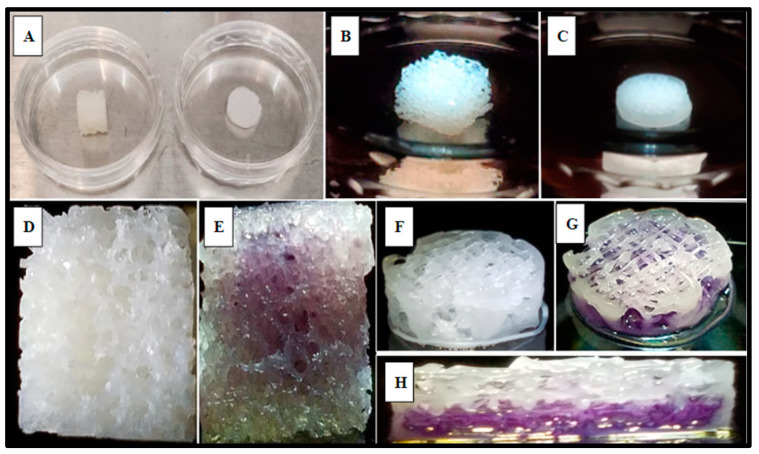
Gross examination of viable SAOS-2 cells integration and localization into scaffold matrices after 5 days via MTT whole scaffold staining. Panel (**A**) displays representative Allowash^®^ (**left**) and PCL scaffold (**right**) before SAOS-2 seeding and culture. Panels (**B**,**C**) show seeded scaffolds in culture. Panels (**D**,**F**) display seeded scaffolds before MTT whole scaffold immersion. Panels (**E**,**G**,**H**) display seeded scaffolds following whole scaffold MTT immersion for 1 h. Formazan crystal formation (purple) can be seen within the scaffold matrices marking the location of viable cell populations. Panel (**H**) shows a full-thickness cross-section of the PCL scaffold with uniform viable cell distribution seen in the basal levels of the scaffold matrix.

SAOS-2 cells were also cultured directly into the 3D-printed PCL material using the rectilinear infill pattern and compared to the allograft Allowash^®^ cancellous bone void filler via MTT whole scaffold staining (Methods, Section 4.4). Following the culture period of 5 days with one full media change at 72 h, the scaffolds were visualized for cell incorporation and localization. The viable cell population embedded within the scaffold matrices reduced the MTT salts to yield purple formazan crystal formation, which was examined grossly (Figure 5) to demonstrate the extensive cell infiltration and high viability in both the PCL fabricated scaffolds and the Allowash^®^ control matrix. These findings support that the PCL material allowed for cell infiltration and attachment to the substrate comparable to bone void fillers currently used in the clinical setting.

**Figure 6:**
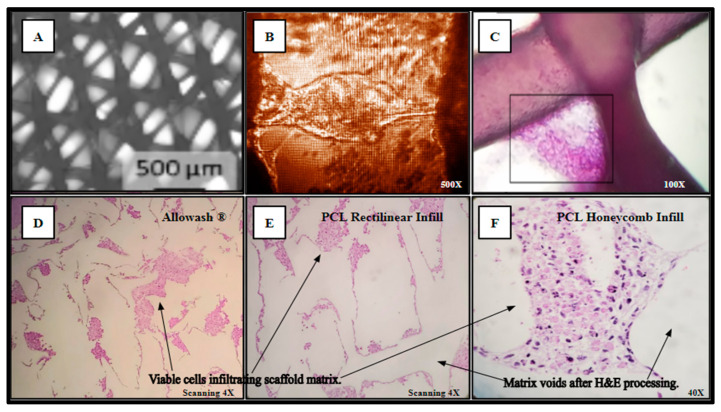
(**A**–**F**) Cell-scaffold infiltration and integration demonstrate a lack of detectable cytostatic or cytotoxic activity in culture with SAOS-2 representative cell line.

Separately inoculated scaffolds from the same cohort were harvested for histological analysis and processed as outlined in (Methods, Section 4.10) yielding Hematoxylin and Eosin (H&E) staining of the viable SAOS-2 cell population occupying the scaffold matrix (Figure 6). In panel A, the PCL scaffold matrix is shown before inoculation. An inverted light micrograph of living cells bridging intra-scaffold space (5 days monolayer culture) is shown in panel B. The H&E stain image of a thick-seeded PCL scaffold infill layer without routine histologic processing to maintain PCL structural components with associated cells (5 days monolayer culture) is in panel C. The seeded Allowash^®^ allograft cube H&E stain following decalcification and routine histologic processing (5 days rotating shaker culture) shows less cells stained in panel D than the PCL seeded scaffolds H&E stains following routine histologic processing (5 days rotating shaker culture) (Panels E, F).

The resulting slides were reviewed by multiple board-certified pathologists, who determined that the cells within the scaffold matrix did not display any microscopic evidence of apoptosis or necrosis, with uniform cell deposition and adhesion that was observed throughout all the examined fields. Clear demarcation can be seen between the deposited layers of viable cells and the scaffold matrix voids with no evidence of reactive basal layer formation seen as intermediate between the cell populations and acellular matrix voids. Uniform cellular viability was noted throughout the sectioned planes of the scaffolds and no cytological evidence of hypoxic necrosis or reactive inclusions were identified. Histological examination of the cell-scaffold interaction at the cellular level provided conclusive evidence of the lack of reactive changes and the occurrence of true cell anchoring to the PCL substrate.

### 2.5. Investigation of Varying 3D-Printed PCL Scaffold Matrix Patterns on Cell Integration and Resulting Scaffold Biomass Increase In Vitro with Primary Human Bone Marrow Cell Culture as Compared to Standard-of-Care Allograft Cancellous Bone Cubes

**Figure 7:**
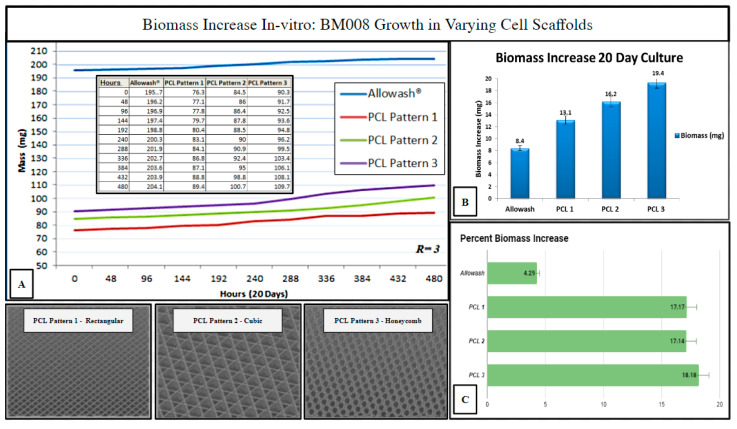
Biomass increase in varying infill pattern 3D printed PCL-scaffolds (*n* = 3 each) as compared to Allowash^®^ cancellous bone cubes (*n* = 3). Panel (**A**) shows the averages of total seeded scaffold mass (mg) measured over the study period at 48-hour intervals. Panel (**B**) displays each test group’s average biomass increase (mg). Panel (**C**) displays the average % biomass increase in each test group. The PCL Pattern-3 (Honeycomb) was selected for further studies based on this infill pattern’s superior % biomass increase.

Three PCL scaffold matrix infill patterns were chosen to compare to Allowash^®^ cancellous bone cubes for (hBM) in vitro biomass increase to measure overall cell-scaffold integration (Methods, Section 4.9). The 3D PCL scaffolds outperformed the cancellous bone Allowash^®^ cubes with regards to overall biomass increase (milligrams) when seeded with (hBM) cell line BM008 during the study period of 20 days, with the honeycombed pattern being identified as being superior to the rectangular and cubic infills (Figure 7).

The measured biomass increase was used to identify the 3D-printed infill pattern most suitable for primary hBM cellular integration which is dependent, in part, on scaffold pore size and matrix density. The ideal infill pattern should display superior biomass increase resulting from the matrix density being favorable for initial cell retention while still allowing for acceptable nutrient perfusion and diffusion of cellular waste products. The honeycomb infill pattern was selected as the pattern of choice for further immunohistochemical studies as it allowed for superior cell growth and biomass increase (Figure 7). The evaluation of the impact of scaffold micro-architecture with regards to intra-scaffold population growth was important to ensure that adequate biological material would be present for the required histopathologic and immunohistochemical studies. In addition, the identification of a superior micro-architecture/infill pattern of the fabricated scaffolding for the purposes of promoting primary (hBM) cell growth will allow for future optimization of tissue scaffolding development for this particular setting.

### 2.6. Histologic Analysis of Human Bone Marrow (hBM) Stem Cell Viability, Integration, and Differentiation in 3D-Printed PCL Honeycomb Scaffold

**Figure 8:**
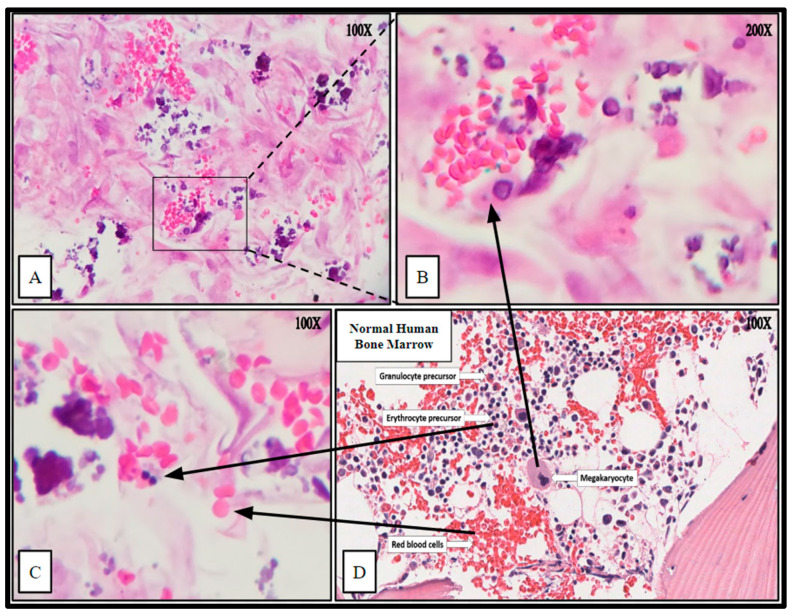
Hematoxylin and Eosin (H&E) staining of PCL scaffolding seeded with hBM cell population BM008 and cultured for 21 days in rotating shaker culture. Panels (**A**–**C**) demonstrate de-novo erythropoiesis and differentiation of the hematopoietic subset of the cultured primary hBM cells. Panel (**D**) is a representative image of normal human bone marrow. Arrows indicate histologic and cellular components observed in the seeded scaffolds and their corresponding presentation in the human bone marrow.

**Figure 9:**
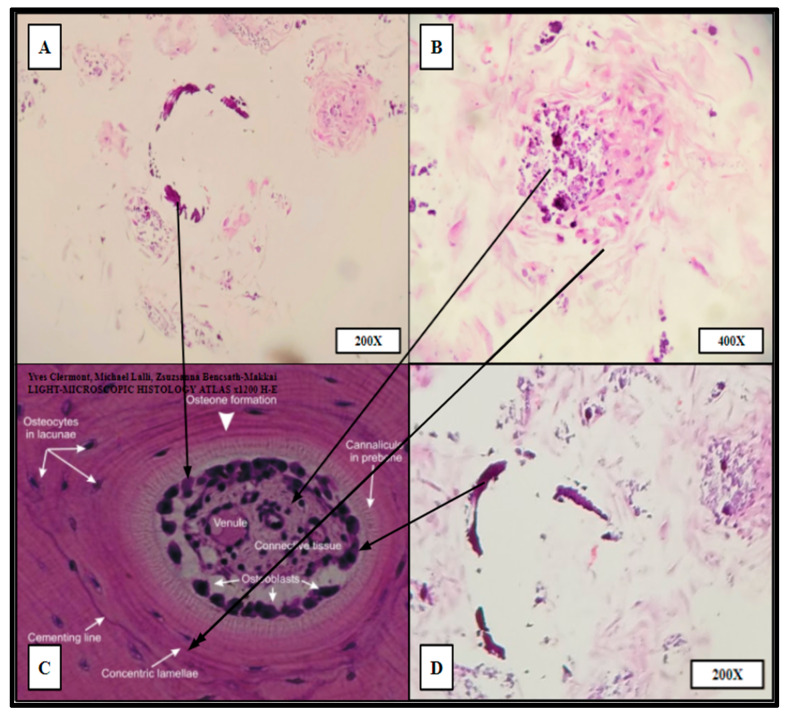
“Proto-osteon” cellular arrangement observed by routine H&E staining of HC infill pattern 3D printed PCL scaffolds seeded with primary hBM cells and cultured for 21 days in rotating shaker culture. Panels (**A**,**B**,**D**) display in vitro de novo histology resembling cellular arrangement and specialization similar to native mature bone architecture (Panel (**C**)). Partial rings of mineralization (dark purple crescents in **A**,**D**) can be seen within the cultured scaffold as well as concentric organizations of stromal-like cells (**A**,**B**,**D**). Panels (**B**,**D**) were imaged from HC infill PCL scaffold seeded with BM008. Panel (**A**) was imaged from HC infill PCL scaffold seeded with BM009.

The impregnated (hBM) scaffolds were processed (Methods, Section 4.10) by routine formalin fixation and paraffin embedding (FFPE). The resulting FFPE samples were sectioned by microtomy and stained with Hematoxylin and Eosin (H&E) to examine the histology and cellular architecture of the fertilized scaffolds. The H&E stains yielded cellular arrangement and components highly analogous to native human bone marrow (Figure 8 and Figure 9).

The cytologic characteristics and cellular organization observed were analogous to the in vivo bone marrow niche with a comparable level of heterogeneity of cellular phenotypes and mineral deposition. Specifically, cell types consistent with megakaryocytes and nucleated erythrocytes were noted, as well as mature erythrocytes that were not present at the time of scaffold seeding (Figure 8). Notably mature erythrocytes, nucleated erythrocyte precursors, megakaryocyte-appearing cells, stromal components, and microcalcifications can be seen in the seeded PCL scaffolding. Before seeding the scaffold, the hBM cell population was completely cleared of all non-nucleated cells and mineral components.

Ring-like mineral deposits were distributed throughout the populated areas of the scaffold with associated concentric stromal cells, which resembled osteon formation; these were termed “proto-osteons” (Figure 9). The H&E staining strongly indicated that the 3D-printed PCL Honeycombed scaffold provided an appropriate structural analogue free of any biologically active additives, which allowed and promoted the generation of cancellous-like de novo bone from primary bone marrow stem cells.

**Figure 10:**
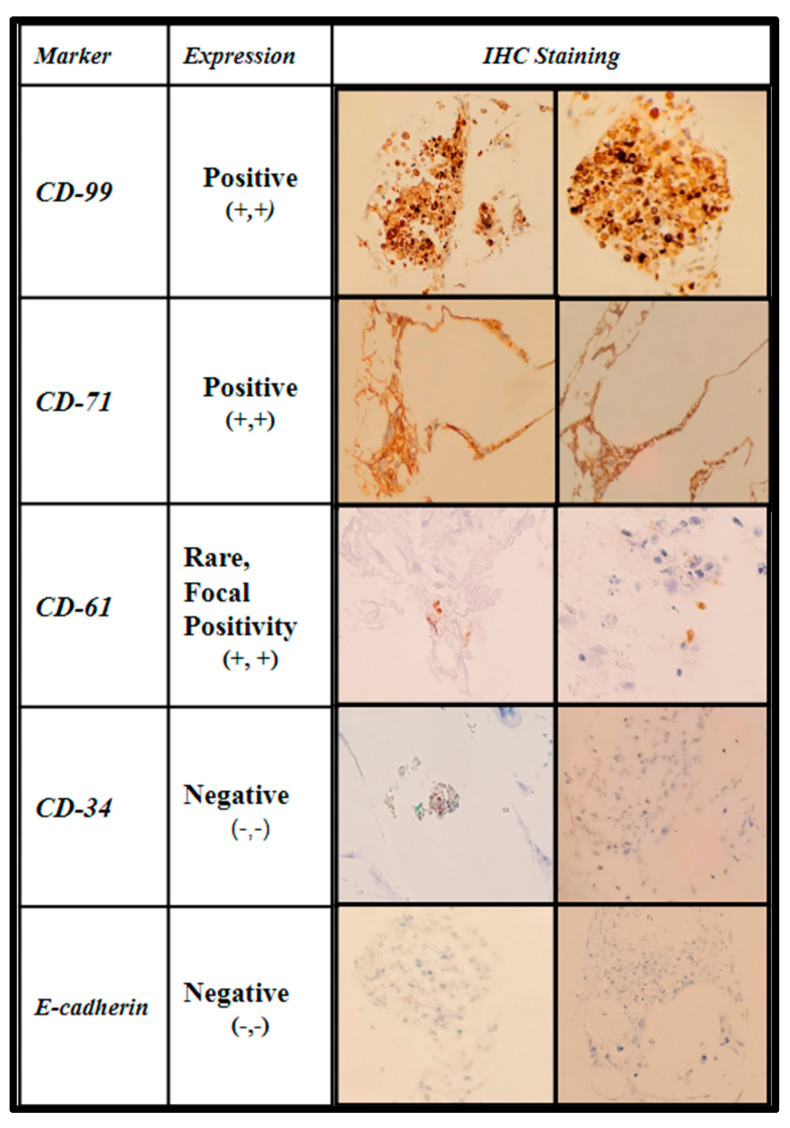
Immunohistochemical analysis of immature human bone marrow markers CD-99, CD-71, CD-61, CD-34, and E-cadherin of primary (hBM) seeded honeycomb infill PCL scaffold cultured for 21 days in rotating shaker culture.

**Figure 11:**
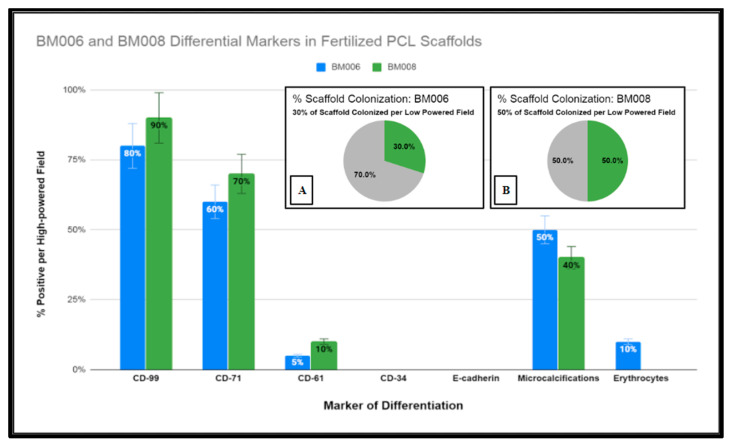
Graphical representation of immunohistochemical studies performed of immature human bone marrow markers CD-99, CD-71, CD-61, CD-34, and E-cadherin of primary hBM seeded honeycomb infill PCL scaffold cultured for 21 days in rotating shaker culture. Insets (**A**,**B**) display percentages of PCL scaffolds adequately colonized by (hBM) cell populations BM006 and BM008 for histological analysis.

The samples were further stained via immunohistochemistry (IHC) to characterize the level of maturation of the seeded BM cells using CD-99 (*cluster of differentiation marker-99*), an early regulatory marker seen in pre-B cell lineages and granulocyte precursors; CD-71 (*transferrin receptor 1*), known to be present in early erythroblast precursors as an integral protein for iron utilization, and a selective marker for adipose and bone marrow-derived MSCs; CD-61(*integrin β3*) an additional stromal cell marker; CD-34 (*Sialomucin*), a selective hematopoietic stem-cell marker, and E-cadherin (*epithelial cadherin*)*,* a specific marker for erythroid differentiation. The IHC expression data are displayed in Figure 10 and Figure 11.

The first two columns are shown in Figure 10, displaying the IHC marker (first column) and the interpretation per high-powered field (second column). The third and fourth columns show images of the staining in representative fields.

Due to the fixed culture period of 21 days used in this study for the histological examination of the seeded scaffolds, only a percentage of the scaffold matrices were colonized at the time of examination. The percentage of the scaffold matrix colonized by the active primary hBM cell lines BM008 and BM006 that were of sufficient density for analysis are displayed in Figure 11. The level of positivity for the selected markers was assessed by board-certified pathologists and estimated rates of positive cells were scored as the percentage of positive cells per high-powered field. In addition, the prevalence of structural components (mineralization) and cellular components (erythrocytes) were also scored in the same fashion by viewing the corresponding H&E slides from each sample.

In combination, the H&E and IHC results demonstrated both the integrity and viability of the intra-scaffold cellular components as well as the ability of the scaffold matrix to support the growth and differentiation of diverse cellular lineages found in the native bone marrow without the addition of exogenous stimulation. However, given the primitive level of the cell lineages examined in this study, all elements could not be definitively characterized either with staining or morphology and further in vitro and in vivo studies may be useful to this end.

## 3. Discussion

As reported previously, scaffolding material acceptable for the effective healing of bony defects must meet a variety of criteria for osteoinductive and osteoconductive potential. This study focused on the limited scope of evaluating the fitness of a 3D-printable material, thus potentially customizable to the patient’s needs, that is biocompatible for use as a bone void filler for orthopedic and or maxillofacial reconstruction. 

The 3D-printed PCL “honeycomb” infill pattern cell scaffold demonstrated properties suitable for hBM stem cell integration, growth, and differentiation. The presence of a diverse population of native bone marrow cell types with the associated microenvironment architecture in conjunction with expected differentiation products indicate that the PCL scaffolding allows for whole hBM stem cell populations to thrive without detectable selection for specific progenitor cell lineages in the *absence of exogenous additives*. The findings of the study indicate the potential for the 3D-printed PCL scaffolding material serves as a more than adequate bone void filler with osteoinductive and osteoconductive properties while preserving functions of native cancellous hematopoietic activity.

When compared to allograft bone void fillers derived from allogenic sources, the 3D-printed PCL scaffolding material is more cost-effective and poses less risk of disease transmission. In addition, it has improved shelf-life and improved customizability over currently used products. Although autologous bone grafting is still the standard for defect-filling applications in bone reconstruction, autograft availability in large defects can be challenging. The 3D-printed PCL scaffolding matrix examined in this study was found to have significant properties allowing it to potentially function as an independent bone graft, even without prior seeding, circumventing the limitations of allograft fillers while demonstrating outstanding osteoconductive and inductive properties in a stand-alone manner. Furthermore, the potential for further enhancement by seeding these fabricated scaffolds with harvested hBM holds the potential for enhancing the timing of incorporation, and remodeling of large defects.

To date, a wide variety of materials and preparations are currently being examined to achieve similar results; however, a significant majority of the published work relies on materials with impregnated bioactive compounds [27,50]. The bioactive nature of these materials may pose additional regulatory requirements for their eventual clinical use as they could be classified as therapeutics in addition to medical devices. In contrast, the 3D-printed PCL scaffolding material described in this study is acellular and absent of bioactive molecules. Instead of chemical or hormonal induction of cellular proliferation and differentiation, the 3D-printed PCL scaffolds rely purely on hBM stem cell organization within the matrix to facilitate cellular signaling and subsequent auto-differentiation and self-organization via innate cellular signaling.

The demonstrated ability of hBM stem cells to self-organize and proliferate within the 3D-printed PCL material coupled with the observation of preserved cellular heterogeneity and differentiation indicates that the novel material is on par with, if not superior to, allograft materials. 

Based on our findings, we believe that future in vivo animal studies of the 3D-printed PCL scaffolding material are warranted. These future studies will allow a comprehensive analysis of the suitability of the material to facilitate bone healing and the exploration of using advanced imaging techniques coupled with 3D printing technology to generate custom bone void scaffolds for improved outcomes of orthopedic and maxillofacial reconstructive surgery at decreased costs. Finally, one of the limitations of this study is the relatively low rate of PCL biodegradation, which will be addressed as part of the authors’ future studies.

## 4. Materials and Methods

**Figure 12:**
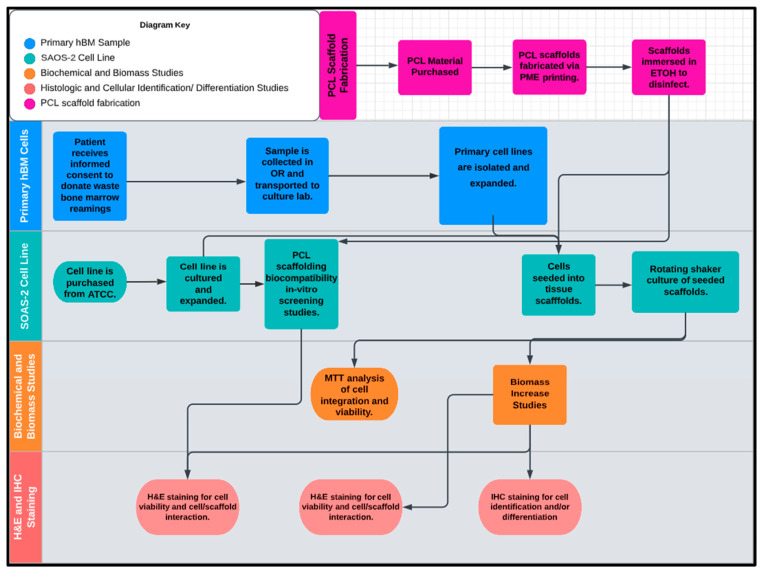
The proposed workflow of this study included five inter-related phases: (1) PCL scaffold microfabrication, (2) primary hBM sample, (3) SAOS-2 cell line, (4) biomechanical and biomass studies, and (5) histologic and cellular identification (differentiation studies).

The workflow of this study is illustrated in Figure 12, divided into five inter-related phases with the design and fabrication of the PCL scaffolding material occurring first. Once the scaffold test groups were created, the PCL scaffolds were screened for biological activity and compatibility with the full characterized osteogenic cell line model SAOS-2. Following the successful screening for biotic compatibility, the scaffolds were tested for their osteoinductive/osteoconductive properties using primary hBM cell populations derived from patient bone marrow samples retrieved from orthopedic trauma repair. At various concurrent times throughout the study period sample representatives of the seeded scaffolds and primary cell lines were tested and imaged via the experiments detailed in the methods section and the data was cataloged and analyzed. The final endpoints of the investigation were the histologic and immunohistochemical review of the inoculated scaffolds by board certified pathologists. The synthetic bone marrow tissue generated in vitro was judged based upon its similarity to native human bone marrow with regards to structural features, protein expression, cellular arrangement, and differentiative cell products of mineralization and erythrocyte production.

### 4.1. Human Bone Marrow (hBM) Sample Collection

Human bone marrow samples were obtained from four patients undergoing intramedullary nail fixation for closed femoral shaft fractures. Patients were selected based on their injuries and were enrolled in an IRB protocol (IRB protocol# 393960) at Marshall University. The study was conducted following the ethical principles provided by the Declaration of Helsinki and the principles of good clinical practice (GCP).

Human bone marrow samples were collected using either the RIA and filtration system for BM sample collection or the standard reaming techniques used to prepare the canal for intramedullary implants. Using these two techniques, whole bone marrow adherent progenitor cell populations including hematopoietic and mesenchymal stem cells were collected and transported to the cell culture lab without any added fixatives or anticoagulants.

### 4.2. Patient Samples of Primary Bone Marrow (hBM) Stem Cell Colonies

BM006, BM008, and BM009 were collected as bone marrow aspirates from femur fractures not requiring debridement (open fractures) undergoing intramedullary nailing. The samples were collected in the operating room and transferred directly to the tissue culture laboratory at ambient temperature without additive fixatives, anti-clotting agents, or preservatives. The transfer time from the patient to tissue culture was minimized to less than 3 h to preserve optimal viable cell retrieval. Sample BM007 was collected under the same protocol; however, it was excluded from the study due to insufficient cell viability and low in vitro growth rate due to unknown factors.

### 4.3. Cell Lines and Growth Medium

SAOS-2 (human osteogenic sarcoma) cell line was purchased from Sigma Aldrich (St. Luois, MO, USA) to serve as a benchmark representative cell culture to assess 3D-printed PCL scaffold biocompatibility and to determine optimal scaffold matrix design. SAOS-2 is the most commonly used standard in investigations of osteoinductive and osteoconductive properties of fabricated tissue scaffolds in vitro. The SAOS-2 (Saos-2 cells, ATCC ^®^ HTB-85) cell line was derived in 1973 from an 11-year-old Caucasian female with osteosarcoma displaying osteoblastic features. The SAOS-2 cell line is often used as a model in osteo-regenerative studies due to its propensity to produce mineralized matrices in culture. The SAOS-2 cells were cultured in a basal liquid medium of RPMI-1640 (Thermo-Scientific, Waltham, MA, USA) supplemented with 10% Human AB Serum (Valley Biomedical). The culture conditions for the SAOS-2 cells and the primary hBM cell lines were consistent to allow for a direct comparative study of the various cell lines. The SAOS-2 cell line was maintained and subsequently passaged throughout the duration of the study using Accutase^®^ (Thermo-Fisher), a non-enzymatic cell detachment solution. 

### 4.4. Cytotoxic and Cytostatic Analysis of PCL Extract on Established Osteosarcoma Cell Line SAOS-2 via MTT Analysis

Polycaprolactone (PCL) 3D-printed tissue scaffolds consisting of 10 mm × 5 mm disk-like samples were incubated in basal RPMI-1640 cell culture medium supplemented with 10% Human AB serum for 120 h at 37 °C to yield PCL medium extract solution to evaluate the cytotoxic and cytostatic potential of fabricated PCL tissue scaffolding material on human cells. SAOS-2 cells were counted (Methods, Section 4.6) and plated into 96-well tissue culture-treated microplates (Thermo-Fisher Scientific) at a density of 5 × 10^3^ cells/well. Treatment groups (*n* = 8) of SAOS-2 cells were cultured in the PCL extract medium for 48 h and then assayed for viability and cell growth. A colorimetric MTT (3-[4, 5-dimethylthiazol-2-yl]-2, 5 diphenyl tetrazolium bromide) assay was used to measure in vitro the cytotoxic and cytostatic effects of PCL extract medium on the SAOS-2 cell line. MTT (Acros Organics) was dissolved in PBS to create a 0.5 mg/mL MTT solution and 100 µL of this solution was added to the wells of a 96-well plate (Thermo-Fisher Scientific). The plates were then incubated at 37 °C for 4 h in 5% CO_2_. After incubation, 50 μL DMSO was added to all the wells, and plates were read on a VersaMax^®^ plate reader (Molecular Devices) at a wavelength of 450 nm to determine the viability of the treatment groups relative to a non-PCL extract control group.

**Figure 13:**
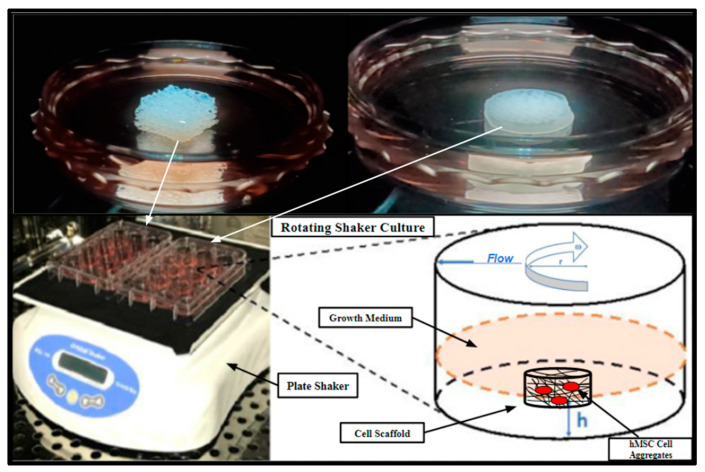
Rotating shaker culture of fertilized PCL scaffolds and Allowash^®^ cancellous cubes at 5 rpm to allow for culture medium flow through the scaffold matrices. The top two images are of the cancellous bone cubes (**left**) and PCL honeycomb scaffold (**right**). The bottom left is a photograph of the rotating shaker culture apparatus (VWR Microplate Shaker), and the bottom right is a schematic demonstrating the desired culture conditions.

SAOS-2 cell cultures were also directly seeded into 3D-printed PCL scaffolds and Allowash^®^ cancellous bone cubes at a density of 1 × 10⁶ cells per scaffold and cultured for 5 days in a rotating shaker culture (Figure 13) with a complete media change at 72 h. The scaffolds were then harvested and submerged in MTT (Acros Organics) 0.5 mg/mL solution for 1 h to allow for gross examination of cell viability and localization within the scaffolding matrices.

### 4.5. hBM Stem-Cell Culture and Colony Expansion

After collection, the whole marrow samples were processed by mechanical homogenization via manual dissociation with sterile scalpel dissection and shear stress dissociation via vortex treatment and immediately transferred to 10 cm tissue culture treated Petri dishes (Thermo Fisher Scientific) and cultured in basal growth medium RPMI-1640 (Thermo-Fisher Scientific) supplemented with 10% Human AB (HS) serum (Valley Biomedical) at 37 °C in 5% atmospheric CO_2_ in high-humidity water jacket tissue culture incubators (Heracell Thermo Scientific).

The samples were monitored using an inverted light microscope (Zeiss) and washed daily with sterile saline to remove non-adherent cell populations and debris including erythrocytes. Fresh growth media was added, as necessary. The adherent cell populations were sub-passaged using cell detachment solution Accutase^®^ (Thermo Fisher Scientific) to expand population size for subsequent imaging, H-E staining, spheroid culture, and scaffold inoculation. The in vitro growth rates of the samples were monitored using Trypan blue exclusion cell counting on the Nexcelom Mini^®^ cell counter (Methods, Section 4.6) during the culture expansion phase and before scaffold seeding.

### 4.6. Cell Counting

Cells were dissociated using Accutase^®^ (ThermoFisher Scientific) and counted using Trypan blue exclusion method and a Cellometer-Mini Automatic Cell Counter (Nexcelom). Cell counting was performed before the seeding of scaffolds and test groups used in all the additionally described studies.

### 4.7. Scaffold Inoculation and Rotating Shaker Culture

3D-printed PCL scaffolds with honeycomb, cubic, and rectangular infill patterns (n = 3 of each group) along with a control group of Allowash^®^ cancellous bone cubes (n = 3) were seeded with the primary hBM stem cell cultures BM006, BM008, and BM009. The cells were seeded in a single-cell suspension at a density of 1 × 10⁶ cells per scaffold, equilibrating to a cell density of approximately 4200 cells per mm³, respectively. The seeded scaffolds made it possible to rest submerged in media for 12 h in non-tissue culture-treated 60 mm Petri dishes under standard tissue culture conditions to allow for initial cell attachment to the scaffold matrices before shaker culture. After the 12 h cell attachment period, the inoculated scaffolds were placed on a rotating shaker and further cultured in 60 mm non-tissue culture-treated Petri dishes for 21 days with complete media changes occurring every 72 h (Figure 13). These scaffolds were used for biomass increase studies and further immunohistochemical studies to assess integration, viability, and cell differentiation. Rotating shaker culture was also utilized for the biocompatibility screening performed with the SOAS-2 cell line and then replicated with the primary hBM cell cultures (Results, Section 2.4). Rotating shaker culture was used in this study to allow for nutrient perfusion throughout the scaffolding matrices to support cell integration and growth while avoiding cellular waste accumulation and to limit cell apoptosis resulting from possible hypoxia anticipated within the innermost areas of the 3D structure, which might result in static media conditions (Figure 13). Rotating shaker culture was conducted with basal growth medium RPMI-1640 (Thermo-Fisher Scientific) supplemented with 10% Human AB (HS) serum (Valley Biomedical) at 37 °C in 5% atmospheric CO_2_ in high-humidity water jacket tissue culture incubators (Heracell Thermo Scientific).

### 4.8. PCL Scaffold Microfabrication and Specifications

Polycaprolactone (PCL) material derived from caprolactone monomer via ring-opening polymerization was purchased from Cellink (Boston, MA, USA). The PCL powder was loaded into the printer cartridge (as received) and maintained at 120 °C. The liquefied PCL material was injected into the printing head using compressed air, resulting in pressure-driven material deposition on a free surface via a converging conical micro-capillary action. To ensure steady-state and uniform material transport in the PME process, the loaded PCL was kept in the heated barrel for three hours before material deposition. The flow pressure was set to 550 kPa (supplied by an oilless, rust-free air compressor). Laminar molten PCL flow was deposited on a glass surface (steadily and uniformly kept at 45 °C) with a print speed of 0.35 mm/s, using a 200 μm nozzle. A porous, cylindrical scaffold was designed, having a diameter and height of 10 mm and 3 mm, respectively. Fabricated with a layer height and width of 150 μm, the scaffolds were printed with rectangular, cubic, and honeycomb infill patterns, respectively. The post-deposition rate of material solidification and layer adhesion was controlled using the chamber fan operating system with a load of 100%.

### 4.9. In Vitro Biomass Increase

Single-cell suspensions of the primary hBM cell line BM008 were injected directly into test groups of 3D-PCL scaffolds fabricated via PME with honeycomb, rectangular, and cubic infill (n = 3) of each pattern along with a control group (n = 3) of Allowash^®^ cancellous bone cubes. Before culture inoculation, each scaffold was hydrated with a culture medium and weighed on an analytical balance (Mettler Toledo, Columbus, OH, USA). The initial mass of each scaffold was recorded and then the experimental group was inoculated with BM008 at a density of 1 × 10⁶ cells per scaffold equilibrating to a cell density of approximately 4244 cells per mm³ per scaffold. The inoculated scaffolds were cultured via rotating shaker culture in 6-well non-tissue culture treated culture plates, one scaffold per well, as previously described (Methods, Section 4.7) for 21 days with each inoculated scaffold being reweighed at 48-hour intervals to record the biomass increase within the scaffolds. The culture media for each test group was changed every 72 h.

### 4.10. Histologic Processing: H&E and Immunohistochemical Evaluation of Cell Viability, Cell Integration, and Cell Differentiation of Primary (hBMs) in Fertilized Scaffolds

Following the 21-day rotating shaker culture as previously described in (Methods, Section 4.6) the seeded tissue scaffolds were harvested and placed in 10% (NBF) neutral buffered formalin (Leica Biosystems, Wetzlar, Germany). The test groups were left in formalin for complete fixation for 24 h. The Allowash^®^ cancellous cubes were briefly decalcified before processing using Formical^®^ solution (Stat laboratory). After thorough fixation and decalcification, the samples were transferred to histological cassettes and processed via routine pathological tissue processing in a Tissue-Tek VIP tissue processor (Sakura) for immunohistochemical analysis. The samples were processed using a routine biopsy processing protocol (Sakura Finetek, Alphen aan den Rijn, The Netherlands). The processed samples were then embedded in paraffin for microtomy sectioning at a thickness of 5 µ and representative slides of each sample were stained via standard H&E (Sakura Tissue-Tek Prisma). Additional slides were generated and stained via IHC for the following markers; CD-99, CD-71, CD-34, and CD-61 (DAKO Omnis, Agilent, Santa Clara, CA, USA).

### 4.11. Sterilization of 3D-Printed PCL Scaffolds and Allowash^®^ Cancellous Cubes

Before any in vitro studies, the various tested scaffolding materials were immersed in 100% ETOH (Sigma Aldrich) for 24 h followed by a thorough rinse and submersion in deionized distilled water. Due to the melting point of the PCL material being approximately 60 °C, sterilization by autoclave was avoided. The Allowash^®^ cancellous bone cubes were supplied pre-sterilized; however, they were subjected to identical ETOH treatment protocols to eliminate any additional confounding variables. 

### 4.12. Statistical Analysis

Statistical analysis was performed for applicable studies using GraphPad Prism 6 statistical software (Graphpad, Inc., La Jolla, CA, USA). One- and two-way analyses of variance (ANOVA) with Tukey or Bonferroni multiple comparisons post test was used to determine the statistical significance of the differences between experimental groups. *p*-values of less than 0.05 were considered statistically significant.

## 5. Conclusions

3D printing of scaffolds for tissue engineering is an area of research in rapid evolution, which produces more defined and biomimetic scaffolds with bioactive factors to enhance their functionalities. A major aim in the development of 3D-printed scaffolds is the creation of scaffolds, potentially customizable to the patient’s needs, that more closely resemble the native microenvironmental properties at the site of implantation, with load-bearing mechanical properties and a microenvironment arrangement that allows nutrient diffusion, cell migration, and the proper environment for the promotion of angiogenesis and/or osteogenesis, that in turn allow the osteointegration of the grafted material. Lastly, fabricating simple yet mechanically robust and dimensionally accurate geometric microstructures with various internal morphologies [11], which allow for rapid osteointegration, are eloquent and simple, and make clinically expedient and meaningful scaffolds. The inherent strength of such geometric structures may also provide for potential enhancement of durability, and although not necessarily the goal for clinical incorporation and remodeling, could be further optimized through independent studies. Consequently, the significant efforts directed at “recreating” the natural random pattern in native bone trabeculae may seem prudent, and certainly is a meaningful endeavor from the standpoint of 3D techniques, may be wholly unnecessary from a biological standpoint [51]. Simply put, human stem cells may not require an exact native architecture but may only require certain geometric constraints such as specific angular surfaces and/or pore size limitations. The implications are obvious and potentially immense for bone scaffold fabrication.

The 3D-printed PCL scaffolding material presented in this study allowed the organization, proliferation, and differentiation within the matrix of diverse cellular lineages found in the native bone marrow without the addition of exogenous stimulation, using Allowash^®^ material as the standard, showing that the PME 3D-printed PCL scaffolding with *simple* geometry is comparable, if not superior, to these allograft materials. Ultimately, PME 3D printing of scaffolds for personalized tissue engineering may be the key to giving those suffering from dysfunction caused by orthopedic and or maxillofacial large bone defects a chance at an improved recovery. Although additional in vitro and in vivo studies are needed to further characterize the 3D-printed PCL scaffolds seeded with hBM stem cells, the fact that these scaffolds can be easily fabricated using PME 3D printing technology illustrates using simple geometric structures to effectively promote human bone osteogenesis is an effective strategy for the meaningful treatment of orthopedic and maxillofacial boney defects.

## Data Availability

The datasets used and/or analyzed during the current study are available from the corresponding author on reasonable request.

## Abbreviations

MTT	4, 5-dimethylthiazol-2-yl]-2, 5 diphenyl tetrazolium bromide
BVF	Bone Void Fillers
Cub	Cubic
DBM	Decellularized Bone Material
DIW	Direct Ink Writing
FFPE	Formalin Fixation and Paraffin Embedding
FDM	Fused Deposition Modeling
H&E	Hematoxylin & Eosin
HC	Honeycomb
hBM	Human Bone Marrow
IC	Immunohistochemistry
ICBG	Iliac Crest Bone Graft
LGDW	Laser-Guided Direct Writing
MSCs	Mesenchymal Stem Cells
PME	Pneumatic Micro-Extrusion
PCL	Polycaprolactone
RL	Rectilinear
3D	Three-Dimensional
SLA	Stereolithography
